# Editorial: Structural and quantitative modeling of synapses

**DOI:** 10.3389/fnsyn.2023.1254416

**Published:** 2023-07-25

**Authors:** Jae Hoon Jung, Noreen E. Reist, Sebastian Doniach

**Affiliations:** ^1^Laboratory of Neurobiology, National Institute of Neurological Disorders and Stroke, National Institutes of Health, Bethesda, MD, United States; ^2^Department of Biomedical Sciences, Molecular, Cellular, Integrative Neurosciences Program, Colorado State University, Fort Collins, CO, United States; ^3^Department of Applied Physics, School of Humanities and Sciences, Stanford University, Stanford, CA, United States; ^4^Department of Photon Science, SLAC National Accelerator Lab, Menlo Park, CA, United States

**Keywords:** synapse, active zone, postsynaptic density, synaptic transmission, segmentation, electron microscopy, light microscopy, simulation

The synapse is a specialized junction crucial for neurons to communicate with their target cells. Major structures characterizing the synapse such as synaptic vesicles, active zones, and postsynaptic density (PSD) have been well known although their organization can vary depending on synapse type and species. Structural studies combined with functional studies provided valuable information extending our understanding of the synaptic structure and function. Active zones along the presynaptic membrane of synapses are characterized by one or more dense aggregates of proteinaceous macromolecules, called active zone material, that are associated with various active zone proteins including calcium channels important for synaptic transmission. A few electron microscopy studies provided evidence that there are stereotypic organizations while other light microscopy studies showed dynamic variation occurring at active zones. Electron microscopy studies have also observed PSDs, large electron-dense protein complexes associated with the postsynaptic membrane opposed to the active zone in excitatory synapses. It is widely known that the PSD contains neurotransmitter receptors and critical elements of postsynaptic plasticity.

Here, Heckmann and Pauli provided results about synaptic vesicles and active zones using single-molecule localization microscopy in the form of direct stochastic optical reconstruction microscopy (dSTORM) of Drosophila neuromuscular junctions supporting activity dependent active zone reorganizations. dSTORM can offer protein specificity and provide spatial resolutions of several nanometers. They showed that it is possible to measure the amount and the distribution of proteins of interest in active zones with dSTORM. They also showed compaction of the presynaptic active zone from imaged subclusters of active zone proteins, Bruchpilot and Rab3 interacting molecule-binding protein, during presynaptic homeostatic potentiation by measuring the size of the subclusters. However, the structural and functional details of the synapse are still difficult to examine. Laghaei and Meriney carried out simulation studies employing a morphological model of the structure and function of neuromuscular transmitter release sites. They focused on the role of presynaptic voltage-gated calcium channels, calcium sensors that control the probability of synaptic vesicle fusion, and the effects of action potential waveform shape on presynaptic calcium entry. Their simulations showed that two distinct types of calcium sensors present at the active zone contribute to synchronous and asynchronous vesicle fusion differently; calcium ion buffers were found to affect the probability of synaptic vesicle fusion, and with repeated motor nerve stimulation at frequencies above 1 Hz, changes in the magnitude of transmitter release were observed.

Using a mechanistic kinetic model of sequential maturation transitions in the molecular fusion complex, Martínez-Valencia et al. reproduced modes of neuromuscular transmission from spontaneous release to facilitation and depression. They used three widely accepted predictions: calcium-dependent forward transitions of vesicles from docked to primed states, reversible transition between pre-priming and priming, and unidirectional fusion and recycling. This simulation study showed that the pre-primed state played a role on preventing exceedingly large amounts of release per impulse that were produced without the pre-primed state.

Postsynaptic densities are closely related to active zones. Jung et al. employed cryo-EM tomography to examine their 3D structural details at nanometer resolutions using postsynaptic densities isolated from adult rat brains. Instead of visual assessment of those postsynaptic densities, they used automatic segmentation tools: automatic segmentation optimization method (ASOM) and 3D watershed segmentation. ASOM was designed to segment structures of interest efficiently while significantly reducing user-dependent variations in segmented structures. With ASOM, each apparently intact PSD was automatically segmented, and with the watershed segmentation method, their substructures were also segmented showing that the size distribution of those substructures, called modules, were mostly similar to each other regardless of application of sonication onto isolated PSD fractions. Furthermore, a few modules were found to be large enough to span the entire thickness of the PSD; those modules were called trans-PSD modules. Interestingly, the frequency of those trans-PSD modules was similar to those reported by several super-resolution light microscopy studies. Their findings support that the PSD is composed of modular structures that are probably basic structural components constituting the PSD.

Gore et al. observed synaptic spinules that are thin, finger-like projections from one neuron, which become embedded within the presynaptic or postsynaptic compartments of another neuron. They used 5 nm isotropic focused ion beam scanning electron microscopy to collect a series of images for constructing volumes of adult male rat's CA1 hippocampus. Their 3D analysis of the volumes of the CA1 hippocampus showed that more than 70% of excitatory presynaptic boutons contained at least one spinule; they also showed that spinule-bearing boutons were 2.5-times larger than non-spinule-bearing boutons and that they contained 60% larger postsynaptic densities. Their findings suggest that spinules may be markers of stronger synapses and fundamental components of excitatory synapses in CA1.

The works in this topic provide key information about the active zone, the PSD, and other structures using various electron and light microscopy techniques, morphological model-based simulation, and automatic segmentation and quantification approaches. We hope that this Research Topic will help encourage further studies to elucidate synaptic structures and their identities and functions.

## Author contributions

All authors listed have made a substantial, direct, and intellectual contribution to the work and approved it for publication.

